# The Mediating Role of Conceptions of Learning in the Relationship Between Metacognitive Skills/Strategies and Academic Outcomes Among Middle-School Students

**DOI:** 10.3389/fpsyg.2018.01985

**Published:** 2018-10-23

**Authors:** Giulia Vettori, Claudio Vezzani, Lucia Bigozzi, Giuliana Pinto

**Affiliations:** Department of Education and Psychology, University of Florence, Florence, Italy

**Keywords:** metacognitive skills/strategies, conceptions of learning, academic outcomes, middle school students, mediation analysis

## Abstract

This study investigated the mediating role of conceptions of learning in the relationship between metacognitive skills/strategies and academic outcomes among middle-school students. The self-report “Learning Conceptions Questionnaire” (LCQ) and “Metacognitive questionnaire on the method of study” (QMS—in Italian) were administered to 136 middle-school students and their academic outcomes were collected. Correlation analyses revealed that within metacognitive skills/strategies only self-assessment was positively correlated with academic outcomes. Mediation analysis indicated that a conception of learning as internal attribution of success and failure was significantly involved as mediator in the relationship between metacognitive skills/strategies and academic outcomes. This study permitted to advance our knowledge about the relationship between metacognitive skills/strategies and academic outcomes and it has opened the way to practical implications.

## Introduction

Identifying the factors affecting student learning outcomes and comprehension of the underlying processes is important in the field of educational psychology. These aims are particularly relevant if we consider that some countries of the Organization for Economic Co-operation and Development (OECD, 2013) show low ranking of their adolescent students' academic outcomes. Low academic performances should not be underestimated because of their repercussions ranging from micro-level, i.e., individual, as closely linked with dropout rates, to macro-level, since students' performances as well as rate of drop-out are indicators to evaluate institutions' quality (Spinath, 2012). The transition from middle school to high school represents an important step for students. As reported in the literature (Anderson et al., 2000), this passage is difficult for most students and especially problematic for some. Students' self-regulatory difficulties appear more evident and affect their academic performance. In a vicious circle, academic failure due to a missing adaptation to the new educational phase can negatively affect the student's representation of self as learner or thinker and his/her motivation to learn (Cleary and Zimmerman, 2004). In order to provide a smoother transition, attention should be paid to factors able to support students before, during, and after. It is important that researchers preventively spend their effort to identify factors able to underpin academic success in the period of age ranging from 11- to 14-years old. Scott et al. (1995) showed how previous low academic outcomes, meant as a lacking academical preparation for the next school level, configured as one of the main risk factors to experience difficulty in the transition from middle to high school.

Literature has identified metacognition as a fundamental component for students' academic outcomes (see, i.e., Bryce et al., 2015). However, the extensive research in this area has produced discordant findings on the relationships between metacognitive skills/strategies and academic outcomes, making it difficult to generalize with any confidence about the nature of the role and the process (Sperling et al., 2004). Thus, the complexity of that process requires the consideration of different cognitive and motivational variables. As suggested by Sperling et al. (2004), the relationship between metacognition and academic outcomes needs to be clarified, including further self-regulatory constructs, such as motivation and conceptions of learning and using different samples. A high priority in education is to clarify the other cognitive and motivational variables involved and to clarify the relationship between metacognitive skills/strategies and academic outcomes.

Before introducing our research, we will present the relevant constructs we predict to influence students' academic outcomes.

### Metacognitive skills/strategies and academic outcomes

Metacognition, defined as the knowledge of how the mind works and the intentional control of cognitive processes (Bruning et al., 2011; Salles et al., 2016), shows a key role in the learning process (Sawyer, 2006; Cornoldi, 2010). This construct of metacognitive skills/strategies implies awareness about ourselves as learners (i.e., our own strengths and difficulties, “*I'm good at the elaboration of information”*), about the task (i.e., understanding the task demands, “*multiple-choice tests require memorization of information”*), the strategies (“*integrate information to prior knowledge helps understanding”*), and the interactions between those (i.e., “*some exam questions require deeper understanding or integration of the material than other kind of tasks*;” Flavell and Wellman, 1977; Bruning et al., 2011). It has been demonstrated that metacognition improve during the pre-adolescence and adolescence (Brizio et al., 2015).

In approaching the studying activity, students use different learning strategies. Through planning (i.e., deciding the time to dedicate to a task, strategies to use, planning of activities), monitoring (i.e., awareness of the state of learning process), and evaluation (i.e., personal judgment about learning results) of the learning activity, it is possible to assume an active and autoregulative role (e.g., regulate attentional processes; Zimmerman, 2000). For example, student regulatory strategies are directed toward the management of time (e.g., study schedule) and study environment (e.g., choosing the right place to concentrate on coursework). This includes collaborative activities such as the involvement of others in learning (see, i.e., peer learning) and seeking help from peers and instructors. Students differ in mastering metacognitive skills and strategies and these differences influence thinking and learning processes (Nelson, 1996). Researchers share the idea of conceiving metacognitive knowledge and skills as important variables for the comprehension of learning, alongside, emotions, and motivation (Efklides, 2011). In fact, in the literature the topic of learning strategies deals with the investigation of deep and surface learning (Dinsmore and Alexander, 2012). A relationship between metacognitive skills/strategies and academic outcomes has been found in both a positive and a negative sense. These studies informed us that some types of learning strategies tend to relate better with achievement than others. Specifically, students' use of strategies aimed at seeking meaning and relating ideas led to high quality learning outcomes as compared to students who use unrelated memorizing, proceed to study without purpose, and are driven by fear of failure (e.g., Entwistle et al., 2000; Zeegers, 2004). Students interested in extracting meaning from their learning resources were involved in the use of deep cognitive strategies (Marton and Säljö, 1976). Elaborational processes, critically evaluating knowledge, and relating information to prior knowledge were key points (Trigwell et al., 2005; Loyens et al., 2013). In contrast, students who mainly focus on rote learning and primarily study to pass a test tended to use surface cognitive strategies, such as memorization of information (Trigwell et al., 2005). A further study by Spada and Moneta (2014) was in line with the previous study in showing that maladaptive metacognition was closely associated with surface approach to learning which in turn led to poor academic outcomes.

However, other researchers found very few, mixed, or weak associations between metacognitive strategies and academic achievements. For example, a study of Callan et al. (2017) conducted among 15-year-old students showed few associations since only two metacognitive strategies (i.e., understanding and summarizing) and one learning strategy (i.e., control strategies) were found to relate significantly and positively to achievement. Some students tended to use a greater number of learning strategies that did not relate to achievement, including memorization and elaboration.

A mixed relationship was found by the study of Chan and Lai (2007) conducted with secondary-school students that confirmed a positive relationship between deep learning strategies and academic achievement, but it found no significant relationships between surface strategies and academic achievement.

A weak relationship between metacognitive skills and academic outcomes emerged in the study conducted by Hong et al. (2015) with medical students.

Furthermore, some studies found no relationships between metacognitive skills/strategies and academic achievements. For example, similarly to the results of Sperling et al. (2004) and Kitsantas et al. (2008), the study of Fonteyne et al. (2017) conducted among university students showed that the variable of metacognition failed to contribute to the prediction of academic achievement in all of the study programs considered. Also, Leong and Bettens (2002), in their study on Singaporean students, found an insignificant correlation between academic outcomes and any type of motivation or strategy considered.

In summary, studies have highlighted large discrepancies between metacognitive skills/strategies and academic outcomes (see, i.e., review of Dolmans et al., 2016), perhaps due to a lacking clear theoretical framework, to investigations conducted in a different learning environment, and to different measures of valid tools (see, review of Dolmans et al., 2016), but they also highlighted the complexity of the process that needs to be better clarified. For example, an elaborative learning strategy could be seen as a necessary, but not sufficient condition that will result in improved academic outcomes. As suggested by Sperling et al. (2004), the relationship between metacognition and achievement needs to be clarified, including further self-regulatory constructs, such as conceptions of learning and using different samples.

### Students' conceptions of learning as potential mediators

When Säljö (1976) considered conceptions of learning in Swedish university students, he highlighted their different beliefs and understanding of the process of learning. Several investigations (Marton et al., 1993; Purdie and Hattie, 2002) theorized a hierarchy of the different views of learning that students utilized. The lower level refers to a quantitative/surface modality to conceive learning as memorization and accumulation of information. The upper level refers to a qualitative/deep conception of learning, characterized by the abstraction of meaning and personal change through learning (Biggs and Moore, 1993; Marton et al., 1993; Purdie and Hattie, 2002). There is extensive support for the predictive validity of students' conceptions of learning on academic outcomes (see, Lonka and Lindblom-Ylänne, 1996; Marton and Säljö, 1997; Entwistle et al., 2003; Vermunt, 2005; Ellis et al., 2008). However, it has to be noted that the majority of studies in the literature highlighted a model of conceptions of learning mainly overlapping with the beliefs' domain (Marton et al., 1993; Purdie and Hattie, 2002), without including academic emotions and causal attributions for success and failure.

Recently, the construct of conception of learning has integrated the beliefs' domain to that of academic emotions and causal attribution for success and failure. A multi-dimensional model representing students' personal views of learning and constructs of themselves as learners emerged. Using this multi-dimensional model, a number of researchers have explored the structure of this multi-dimensional model (Vezzani et al., 2018), also in a cross-cultural perspective (Cantoia et al., 2011), the domain specific conceptions of learning (Vezzani et al., 2017), and how these conceptions of learning influence academic outcomes (Pinto et al., 2018). In the context of these studies, conceptions of learning were explored with the Learning Conception Questionnaire (LCQ) showing a good psychometrical validity. The model structure showed by Vezzani et al. (2018) with middle-school students presented the following factorial structure (see Table 1). A further study (Pinto et al., 2018) aimed at testing the predictive validity of conceptions of learning among middle-school students showed that conceptions of learning as a “co-constructive and cultural process” and as a “personal challenge, self-efficacy, and personal growth” were positively related to academic outcomes. In contrast, a conception of learning as “reduction of a deficit through individual effort” was a negative predictor. These findings permitted us to identify a varied range of conceptions of learning able to influence academic outcomes in a positive and negative sense, through the use of a multi-dimensional model of conceptions of learning.

**Table 1:** Descriptive statistics of the factorial dimensions of the QMS, LCQ and academic outcomes in language and literature, foreign language and math: minimum and maximum, mean, standard deviation, skewness, and kurtosis indexes.

	**Source**	**Min**	**Max**	**M**	**SD**	**Skewness**	**Kurtosis**
QMS	1. Motivation to study	1.33	2.44	1.92	0.260	−0.27	−0.36
	2. Organization of personal work	10	21	15.36	2.50	0.16	−0.77
	3. Use of supports	7	18	12.64	2.32	−0.01	−0.08
	4. Active elaboration of scholastic material	8	23	14.75	2.37	−0.07	0.64
	5. Flexibility to study	7	20	13.90	2.30	−0.04	0.01
	6. Participation in classroom	5	15	9.18	1.89	0.22	0.39
	7. Concentration	7	18	11.71	2.25	0.17	−0.41
	8. Selection of principal aspects	5	14	9.687	1.60	−0.12	0.33
	9. Self-assessment	9	19	14.37	2.13	−0.18	−0.07
	10. Strategies of preparation for a test	13	34	23.32	3.83	0.14	0.01
	11. Metacognitive sensitivity	8	23	15.75	2.28	−0.17	0.96
LCQ	12. Learning as co-costructive and cultural process	−1.31	0.84	0.01	0.43	−0.55	0.17
	13. Learning as a reduction of deficit through individual effort	−0.82	0.74	0.02	0.32	−0.25	−0.47
	14. Negative experiences and anxiety	−0.43	0.71	0.01	0.29	0.87	−0.02
	15. Personal challenge, self-efficacy and personal growth	−2.04	1.35	−0.01	0.66	−0.51	0.08
	16. Internal attribution for success and failure	−2.47	1.80	0.03	1.01	−0.25	−0.67
	17. External attribution failure	−1.23	2.72	−0.05	0.96	0.93	0.84
Academic outcomes	18. Language and literature	6	10	7.75	0.94	0.63	−0.14
	19. Foreign language	6	10	7.36	1.08	0.53	−0.36
	20. Math	6	10	7.76	1.07	0.35	−0.41

Theoretically, the relationship between conceptions of learning and academic outcomes has been explained by referring to the power of conceptions to influence learning strategies. Since the basilar works of Martin and Ramsden (1987) and Van Rossum and Schenk (1984) empirical evidence of a relationship between students' conceptions of learning and their learning outcomes has emerged. A relationship also subsequently confirmed by Purdie et al. (1996), Marton et al. (1993), Lindblom-Ylänne and Lonka (1999), Entwistle and Entwistle (2003), and Vermunt (2005). In line with them, if students believe that learning mainly overlaps with memorization then they may be more likely to use their cognitive source to memorize the contents of their text books. These beliefs may limit the level of engagement with the learning process and it may subsequently lead to low performance. If students believe there is only one path to solution, then they may be more likely to give up more quickly or engage in less effort if their first attempt is not successful. In contrast, if students believe that learning is personal growth then they may be more likely to increment their personal engagement in studying, adopt more elaborate learning strategies, and persist when a learning goal is not successfully reached. In the context on the studies above, conceptions of learning function as implicit theories able to induce particular learning behaviors through the use of different learning strategies. However, some authors (Muis, 2007) argue that one may test the reverse process corresponding to the hypothesis that self-regulated learning might influence the development of epistemic beliefs. For example, Pintrich et al. (1993) suggested that the explicit teaching of how to make connections across content areas within that domain allowed them to intervene on students' beliefs about the structure of statistics knowledge. This is in line with the view of self-regulation as cyclical (e.g., Borkowski et al., 2000; Schunk, 2001) rather than sequenced. Muis (2007) suggested considering the relationship between epistemic beliefs and self-regulated learning as reciprocal. Students' own preconceptions about learning, including about themselves as learners, could promote students' reflection on their own metacognitive behaviors, as well as the reverse, their metacognitive behaviors could turn out to activate students' conceptions of learning.

### Rationale for this study

After reviewing the literature, it can be concluded that on one hand, the relationship between metacognitive skills/strategies and academic outcomes is not always straightforward. The influence of metacognitive strategies on academic outcomes tracked in some studies is not confirmed by others. Actually, the lacking predictive link between metacognitive skills/strategies and academic outcomes challenges the autonomous power of the metacognitive apparatus in reaching successful academic outcomes. The divergence highlighted by the literature suggests that other variables may act a mediating role in that relationship. The literature shows how conceptions of learning are linked to academic outcomes both directly (Pinto et al., 2018) and indirectly, through the mediating role of metacognitive strategies (see, Vermunt, 2005). However, following the suggestion of Muis (2007) to intend the relationship between metacognition and epistemological beliefs in a recursive way, conceptions of learning can function as a mediator. In following this reasoning, the ways in which middle-school students approach the learning process could pass through the way they conceptualize learning and their perceptions of themselves as learners.

In this study, we investigated the relationship between metacognitive skills/strategies, conceptions of learning, and academic outcomes among middle-school students. Our sample was chosen for the importance of this period in scholastic development. As emerged in the literature, the transition from middle school to high school represents an important step for students. Sometimes this passage is problematic for some students.

Considering our sample, we decided to use self-report instruments (Fulmer and Frijters, 2009). To test metacognitive skills/strategies in learning, we used the “*Metacognitive questionnaire on the method of study*” (the acronym of the Italian version is QMS; Cornoldi et al., 2001). This choice was driven by its consideration of a large pattern of both metacognitive skills and strategies, including organization of materials, active elaboration and flexibility, and approaches to studying, such as self-assessment and choice of strategies. Furthermore, its items are simple to understand and easily administered.

To assess students' conceptions of learning we used the self-report of Pérez-Tello et al. (2005) as in previous studies (see, Cantoia et al., 2011; Vezzani et al., 2018).

### Aims and hypothesis

The intent of this study was to investigate the relationship between metacognitive skills/strategies, conceptions of learning, and academic outcomes among middle-school students.

Specifically, this study aimed to test in middle-school students:

1. The association between academic outcomes both with metacognitive skills/strategies and conceptions of learning:
2. The predictive model of metacognitive skills/strategies and academic outcomes with conceptions of learning as mediating variables.

Regarding the first aim, considering the controversial nature of the relationship between metacognitive skills/strategies and academic outcomes, we cannot make hypotheses in advance. Furthermore, in line with the literature, we expected a differential impact of the pattern of conceptions of learning on academic outcomes. Specifically, we expected that conceptions of learning characterized by a social-constructive view of learning and personal growth would be positively associated with academic outcomes. In addition, we expected that conceptions of learning characterized by an incremental and quantitative view of learning would be negatively associated with academic outcomes.

Regarding the second aim, the assumption that conceptions of learning are mediating variables in the relationship between metacognitive skills/strategies and academic outcomes may be supported based on studies in literature on the reciprocal effect of self-regulation and epistemological beliefs (Muis, 2007). Specifically, we expected that the ways in which middle-school students approach the learning process could pass through the way they conceptualize learning and their perceptions of themselves as learners.

## Methods

### Participants

One hundred and thirty-six middle-school students were recruited from medium-sized urban middle schools (67 males, M-age 12.57 ± 1.02 years; 69 females, M-age 12.83 ± 0.93 years). Forty-three students were in the 6th grade, 41 in the 7th grade, and 52 in the 8th grade. All students who participated in the study passed the final exam and there had been no indications of delay or learning disorders in the students who participated. Students were from a similar socio-economic status (SES)—medium-low. Following National Law 104/1992 and 170/2010, students with a certified learning disorder and/or disability and foreigners who had been in the country for < 5 years were excluded from the study.

All schools in our sample were part of the public system. Parents and school authorities, as well as the students themselves, consented to participate in the study. All subjects gave written informed consent in accordance with the Declaration of Helsinki. The protocol was approved by the Departmental Ethics Committee, Department of Education and Psychology, University of Florence.

### Procedures

Early in the semester, at the end of September, information and the plan of the study were shared with the classes. In the first 2 month-step (October–November), students' metacognitive skills/strategies in learning and conceptions of learning were assessed via self-report questionnaires. The questionnaires were handed out collectively during regular school hours. During this time, both the researcher and the teacher were available to answer any questions. In a second step, going from the end of June to the end of July, students' academic outcomes were collected. Participants were evaluated in two different moments since researchers were interested in studying the process, meant as the effect of two variables, metacognitive skills/strategies and conceptions of learning, on academic outcomes.

### Measures

#### QMS: metacognitive skills/strategies

Students' metacognitive skills and strategies were measured with the “Metacognitive questionnaire on the method of study” (QMS; Cornoldi et al., 2001). The QMS is a 163-item self-report instrument to be answered on a three-point Likert-scale [scores ranging from (1) “I strongly agree” to (3) “I disagree”]. It consists of 21 subjects, grouped into four categories as follows:

1. *Learning strategies* (*N* = 44 items; A-motivation to study, B-staff organization work, C-study aids, D-active material processing, E-flexibility of study, F-class participation; e.g., “When the teacher assigns work to me, I apply myself only if the materials interest me”).
2. *Cognitive styles of information processing* (*N* = 31 items; G-systematic/intuitive cognitive style, H-global/analytic cognitive style, I-impulsive/reflective cognitive style, L-verbal/visual cognitive style, M-autonomy and personal way of approaching studying; e.g., “If a text brings into question a number of issues, I consider them one at a time.”)
3. *Metacognition and study*ing (*N* = 38 items; N-concentration, O-selection of the main aspects, P-self-assessment skills, Q-preparation strategies to a test, R metacognitive sensitivity; e.g., “While listening to an explanation in class, I also think of other things”).
4. *Attitude toward school and studying* (*N* = 50 items; S-relationship with classmates, T-relationship with teachers, U-school anxiety, V-attitude toward school, Z-allocation, and commitment; e.g., “I find it difficult to ask some of my teachers”).

For this study, we proceeded using the scales named “learning strategies” and “metacognition and studying.” Higher scores indicated higher levels of adaptive metacognitive behavior and approach to studying. The QMS has strong internal reliability.

#### LCQ: students' conceptions of learning

Students' conceptions of learning were measured with the “Learning Conceptions Questionnaire” (LCQ; Pérez-Tello et al., 2005). The LCQ consists of 49 items with statements about knowledge or learning, answered on a five-point Likert-scale [scores ranging from (1) “I strongly disagree” to (5) “I strongly agree”]. Three different sections of the LCQ measured the following constructs in relation to the experience of learning: beliefs (18 items), emotions (17 items), and causal attributions (14 items). The section concerning “beliefs” includes items that investigate opinions about the overall structure of the learning process. This section is inspired by the models described by Bruner (1996), and primarily focused on the active or passive approach of learners and their relations with knowledge. However, the section also integrated the affective-emotional dimension of learning (Pérez-Tello et al., 2005). The section on “emotions” concerns the exploration of personal emotional reactions associated with the learning experience and it is in line with academic emotions set in literature (see, Wang et al., 2017). Coherently with the factorial analyses carried out by Pérez-Tello et al. (2005), this section includes growth and personal change (“I feel that learning is an opportunity for growth and personal change”; see, Marton et al., 1993), duty, transmitted by item 19 (“I'm learning as a duty”), and finally the design for a near future, expressed by item 35 (“I'm learning as the path to success; ” see, Purdie et al., 1996; Klatter et al., 2001).

The section of “attributions” deepens mental conceptions related to the outcome of the learning process, as well as explanations of successes and failures. The section explores the students' points of view concerning their attribution of scholastic failure, the ability to learn from their own mistakes, teachers' attribution of students' success or failure, and students' attribution for success in school situations (Pérez-Tello et al., 2005). The factorial structure of LCQ is reported in Vezzani et al. (2018) and is very similar to the factorial dimensions that emerged among university students (Vezzani et al., 2017). The “beliefs” section pointed out two factor dimensions, namely “Learning as a co-constructive and cultural process” (α = 0.65) and “Learning as a reduction of deficit through individual effort” (α = 0.55). In the “emotions” section, there were two latent dimensions pointed out: “Negative experience and anxiety” (α = 0.83) and “Personal challenge, self-efficacy and personal growth” (α = 0.79). Finally, two factors for the “Causal Attributions” section were extracted: “Internal attribution for success and failure” (α = 0.61) and “External attribution for failure” (α = 0.54).

#### Assessment of academic outcomes

Thanks to teacher report data, academic performance was assessed as the average percentage mark on different subjects. In order to have a more reliable measure of academic outcomes, we decided to collect results in different subjects, such as language and literature, mathematics, and foreign languages. As a general note, final grades are the result of tests, both oral and written, held during the school year and represent a necessary condition for admission to the following year. Grades for student classroom work were assigned a mark out of ten, in which 6/10 is considered a pass.

#### Context of research

In our context of research, the Ministry of Education establishes the aims of the educational process, the subjects, the number of teaching hours, the general criteria for student assessment, etc. The program follows a specific curriculum, as indicated in National Guidelines for the Curricula, and it embraces various disciplines ranging from language and literature, foreign language, an additional foreign language, history, geography, mathematics, science, technology, music, art, and sports science. Periodic and annual assessments focus on student learning processes, including their behavior as well as learning outcomes. Furthermore, the Educational Offer Plan (POF in Italian) of each school defines the method and criteria for assuring that the evaluation is uniform and transparent.

### Data analysis

In the first step, all descriptive statistics (minimum and maximum, mean, standard deviation, skewness, and kurtosis coefficients) for QMS, LCQ dimensions, and academic outcomes (language and literature, foreign language, and mathematics) were carried out, with the normality of distribution assured.

In the second step, in line with the first aim of the present study, we calculated the bivariate and partial correlations between QMS, LCQ, and academic outcomes by conducting Bravais-Pearson linear correlation coefficient. As general criteria, LCQ or a QMS dimension was considered a possible mediator when it was significantly correlated with at least two of the three scholastic grades considered (keeping under control all the factorial dimensions of the other questionnaire), coherently with step 3 of Baron and Kenny's model, which said that the mediator must affect the dependent variable when the independent variable is controlled (MacKinnon, 2002).

In the third and last step, in line with the second aim of this study, several mediational analyses were carried out. According to the results of the correlational analyses, and with respect to the simulation study by MacKinnon (2002) that stressed the necessity of steps 2 and 3 of Baron and Kenny's model (1986), the mediational analyses were implemented with factors of the QMS as independent variables, academic achievements as dependent variables, and the conceptions of learning dimensions as possible mediator. The analyses were carried out via SPSS (version 23). The mediational analyses were implemented by the PROCESS package (Hayes, 2013), i.e., a particular SPSS statistical package.

## Results

In Table 1, the descriptive statistics of all dimensions of QMS, LCQ and the academic outcomes are reported. All variables are normally distributed, so no transformation was necessary to implement the following inferential analyses.

Linear correlations between the QMS and LCQ dimensions and students' academic outcomes are shown in Table 2. The results of the correlational analyses on the QMS revealed a significant relationship only between “Self-assessment” and the academic outcomes in language and literature (*r* = 0.22, *p* < 0.05; Table 2). Regarding the conceptions of learning measured by the LCQ, “Learning as a co-constructive and cultural process” resulted in a positive correlation with academic outcomes in foreign language grades (*r* = 0.21, *p* < 0.05). “Personal challenge, self-efficacy and personal growth” showed a directly proportional relationship with the grades in academic outcomes in language and literature (*r* = 0.27, *p* < 0.01). Finally, only “Internal attribution for success and failure” was significantly correlated with all the three grades, i.e., language and literature (*r* = 0.27, *p* < 0.01), foreign language (*r* = 0.23, *p* < 0.01), and math (*r* = 0.22, *p* < 0.05; Table 2). These results suggest the possible effects of a conception of learning “Internal attribution for success and failure” as a mediator of the relationship between scales of QMS with all the academic outcomes.

**Table 2:** QMS, LCQ, and academic outcomes.

		**LCQ**						**Scholastic achievements**		
	**Source**	**12**.	**13**.	**14**.	**15**.	**16**.	**17**.	**18**.	**19**.	**20**.
QMS	1. Motivation to study	0.07	0.09	−0.01	0.26^***^	0.18^**^	0.09	0.05(−0.04)^a^	0.06(−0.02)^a^	0.04(0.01)^a^
	2. Organization of personal work	0.18^**^	−0.08	0.17^*^	−0.04	0.01	0.03	−0.05(−0.06)^a^	0.01(−0.07)^a^	−0.03(−0.07)^a^
	3. Use of supports	0.13^*^	0.01	−0.07	0.25^***^	0.21^**^	0.02	0.08(−0.01)^a^	0.14(0.07)^a^	0.13(0.09)^a^
	4. Active elaboration of scholastic material	0.17^*^	0.16^*^	−0.06	0.28^***^	0.25^***^	0.07	0.11(0.05)^a^	0.06(−0.01)^a^	0.03(0.02)^a^
	5. Flexibility to study	0.15^*^	0.22^**^	−0.12	0.26^***^	0.29^***^	0.07	0.13(0.04)^a^	0.10(−0.02)^a^	0.05(0.04)^a^
	6. Participation in classroom	0.11	0.09	−0.14^*^	0.35^***^	0.32^***^	0.04	0.08(−0.05)^a^	0.09(−0.04)	0.04(0.02)
	7. Concentration	0.01	0.03	0.36^***^	−0.22^**^	−0.13	−0.07	−0.12(−0.10)^a^	−0.14(−0.10)^a^	−0.08(−0.17)^a^
	8. Selection of principal aspects	0.24^***^	0.21^**^	−0.08	0.22^**^	0.31^***^	0.05	0.02(−0.07)^a^	0.06(−0.07)^a^	0.01(−0.04)^a^
	9. Self–assessment	0.10	0.14^*^	0.08	−0.09	0.07	0.09	0.22*(0.26**)^a^	0.16(0.14)^a^	0.15(0.16)^a^
	10. Strategies of preparation for a test	0.21^**^	0.14^*^	−0.21^**^	0.42^***^	0.42^***^	−0.01	0.11(−0.03)^a^	0.16(0.03)^a^	0.12(0.08)^a^
	11. Metacognitive sensitivity	0.15^*^	0.16^*^	−0.18^**^	0.29^***^	0.17^*^	0.03	0.05(−0.06)^a^	−0.03(−0.01)^a^	0.04(−0.09)^a^
LCQ	12. Learning as co–constructive and cultural process							0.15(0.16)^b^	0.21^*^(0.11)^b^	0.12(0.19^*^)^b^
	13. Learning as a reduction of deficit through individual effort							−0.01(−0.04)^b^	0.01(−0.03)^b^	−0.03(−0.01)
	14. Negative experiences and anxiety							−0.13(−0.03)^b^	0.02(0.06)^b^	−0.01(0.10)^b^
	15. Personal challenge, self–efficacy and personal growth							0.27^**^(0.23^*^)^b^	0.09(0.08)^b^	0.13(0.01)^b^
	16. Internal attribution for success and failure							0.27^**^(0.22^*^)^b^	0.23^**^(0.19^*^)^b^	0.22^*^(20^*^)^b^
	17. External attribution for failure							−0.01(0.02)^b^	0.07(0.01)^b^	−0.01(0.11)^b^

* *p < 0.05*.

** *p < 0.01*.

*** *p < 0.001*.

a *Partial correlations between factorial dimensions of QMS and scholastic achievements (taking under control the dimensions of LCQ) are reported in brackets*.

b *Partial correlations between factorial dimensions of LCQ and scholastic achievements (taking under control the dimensions of QMS) are reported in brackets*.

The results obtained by mediational analyses revealed a similar trend for all the academic outcomes (Tables 3–**5**).

**Table 3:** Summary of direct and indirect effects of the dimensions of the QMS on academic outcomes in language and literature considering the LCQ dimension “Internal attribution of own success and failure” as possible mediator: effect, regression parameter B, standard error B, Student *t*-test and *p*-value.

**Conceptual area**	**Source (X)**	**Effect**	**B**	**SEB**	***t***	***p***
Learning strategies	Motivation to study	X → Y	−0.021	0.298	−0.07	n.s.
		X → M → Y	0.198	0.107	1.85	n.s.
	Organization of personal work	X → Y	−0.016	0.030	−0.54	n.s.
		X → M → Y	−0.001	0.010	−0.06	n.s.
	Use of supports	X → Y	0.013	0.033	0.40	n.s.
		X → M → Y	0.020	0.013	1.62	n.s.
	Active elaboration of scholastic material	X → Y	0.017	0.030	0.56	n.s.
		X → M → Y	0.027	0.012	2.03	0.042
	Flexibility to study	X → Y	0.016	0.035	0.64	n.s.
		X → M → Y	0.036	0.015	2.28	0.023
	Participation in classroom	X → Y	−0.009	0.040	−0.24	n.s.
		X → M → Y	0.050	0.018	2.55	0.011
Metacognition and Study	Concentration	X → Y	−0.043	0.037	−1.16	n.s.
		X → M → Y	−0.008	0.010	−0.75	n.s.
	Selection pf principal aspects	X → Y	−0.043	0.052	−0.84	n.s.
		X → M → Y	0.054	0.022	2.27	0.023
	Self-assessment	X → Y	0.104	0.033	−3.15	0.002
		X → M → Y	0.008	0.013	0.62	n.s.
	Strategies of preparation for a test	X → Y	0.001	0.019	0.05	n.s.
		X → M → Y	0.026	0.010	2.59	0.010
	Metacognitive sensitivity	X → Y	−0.004	0.035	−0.12	n.s.
		X → M → Y	0.026	0.014	1.90	n.s.

*X → Y, direct effect after entering the indirect effect; X → M → Y, indirect effect. No significant result (either indirect or direct effect) were pointed out in mediational models about “Motivation to study” “Organization of personal work,” “Use of supports,” “Concentration” and “Metacognitive sensitivity,” and for this reason their results were removed from the table*.

**Graph 1:**
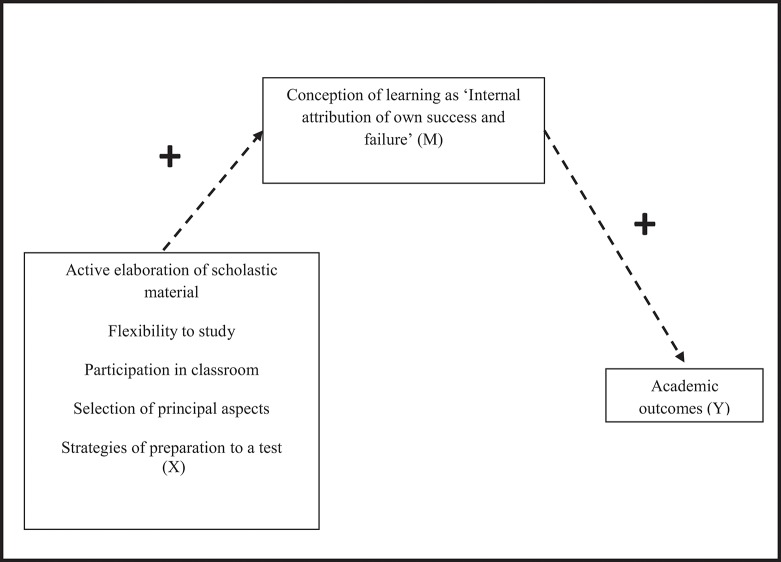
Indirect effects of some factorial dimensions of QMS (Active elaboration of scholastic material, Flexibility to study, Participation in classroom, Selection of principal aspects and Strategies of preparation to a test) on academic outcomes by a conception of learning as internal attribution of success and failure.

Regarding the QMS “*Learning strategies*” section, some factorial dimensions predicted all academic outcomes with a positive correlation that emerged for “Active elaboration of scholastic material” (language and literature: β = 0.027, *p* < 0.05; foreign language: β = 0.027, *p* < 0.05; math: B = 0.028, *p* < 0.05), “Flexibility to study” (language and literature: B = 0.036, *p* < 0.05; foreign language: B = 0.038, *p* < 0.05; math: B = 0.036, *p* < 0.05), and “Participation in classroom” (language and literature: B = 0.050, *p* < 0.05; foreign language: B = 0.050, *p* < 0.05; math: B = 0.047, *p* < 0.05). In the QMS “*Metacognition and Study”* section, we observed a similar positive mediational link (relationship represented in the Graph 1) for “Selection of principal aspects” (language and literature: B = 0.054, *p* < 0.05; foreign language: B = 0.052, *p* < 0.05; math: B = 0.050, *p* < 0.05) and “Strategies of preparation for a test” (language and literature: B = 0.026, *p* < 0.01; foreign language: B = 0.023, *p* < 0.05; math: B = 0.025, *p* < 0.05). The only significant direct linkage relationship emerged for “Self-assessment” (language and literature: B = 0.10, *p* < 0.01; foreign language: B = 0.08, *p* < 0.05; math: B = 0.09, *p* < 0.05; Tables 3–**5**). With respect to the correlation between “Self-assessment” and scholastic outcomes (Table 2), the mediation analyses showed a significant linkage of this dimension also with foreign language and math, after the indirect effect of “Internal attribution for success and failure” had been taken into consideration (Tables 4, 5).

**Table 4:** Summary of direct and indirect effects of the dimensions of the QMS on the academic outcomes in foreign language, considering the dimension “Internal attribution of own success and failure” of the LCQ as possible mediator: effect, regression parameter B, standard error B, Student *t*-test, and *p*-value.

**Conceptual area**	**Source (X)**	**Effect**	**B**	**SEB**	***t***	***p***
Learning strategies	Motivation to study	X → Y	−0.043	0.340	−0.127	n.s.
		X → M → Y	0.188	0.115	1.70	n.s.
	Organization of personal work	X → Y	−0.013	0.034	−0.39	n.s.
		X → M → Y	−0.001	0.009	−0.06	n.s.
	Use of supports	X → Y	0.043	0.037	1.18	n.s.
		X → M → Y	0.018	0.012	1.47	n.s.
	Active elaboration of scholastic material	X → Y	−0.012	0.034	−0.36	n.s.
		X → M → Y	0.027	0.013	2.08	0.038
	Flexibility to study	X → Y	−0.014	0.044	−0.32	n.s.
		X → M → Y	0.038	0.019	2.08	0.038
	Participation in classroom	X → Y	−0.027	0.050	−0.53	n.s.
		X → M → Y	0.050	0.022	2.20	0.028
Metacognition and Study	Concentration	X → Y	−0.033	0.038	−0.87	n.s.
		X → M → Y	−0.008	0.011	−0.73	n.s.
	Selection pf principal aspects	X → Y	−0.048	0.060	−0.79	n.s.
		X → M → Y	0.052	0.025	2.02	0.044
	Self-assessment	X → Y	0.082	0.037	2.18	0.031
		X → M → Y	0.008	0.012	0.60	n.s.
	Strategies of preparation for a test	X → Y	0.010	0.021	0.49	n.s.
		X → M → Y	0.023	0.011	2.09	0.036
	Metacognitive sensitivity	X → Y	−0.006	0.041	−0.15	n.s.
		X → M → Y	0.025	0.014	1.76	n.s.

*X → Y, direct effect after entering the indirect effect; X → M → Y, indirect effect. No significant result (either indirect or direct effect) were pointed out in mediational models about “Motivation to study” “Organization of personal work,” “Use of supports,” “Concentration” and “Metacognitive sensitivity,” and for this reason their results were removed from the table*.

**Table 5:** Summary of direct and indirect effects of the dimensions of the QMS on the academic outcomes in math, considering the dimension “Internal attribution of own success and failure” of the LCQ as possible mediator: effect, regression parameter B, standard error B, Student *t*-test and *p*-value.

**Conceptual area**	**Source (X)**	**Effect**	**B**	**SEB**	***t***	***p***
Learning strategies	Motivation to study	X → Y	0.077	0.363	0.83	n.s.
		X → M → Y	0.193	0.111	1.70	n.s.
	Organization of personal work	X → Y	0.002	0.032	0.07	n.s.
		X → M → Y	−0.001	0.010	−0.06	n.s.
	Use of supports	X → Y	0.045	0.038	1.20	n.s.
		X → M → Y	0.019	0.013	1.49	n.s.
	Active elaboration of scholastic material	X → Y	−0.003	0.035	−0.07	n.s.
		X → M → Y	0.028	0.013	2.15	0.033
	Flexibility to study	X → Y	0.012	0.044	0.27	n.s.
		X → M → Y	0.036	0.019	1.99	0.047
	Participation in classroom	X → Y	−0.000	0.050	−0.000	n.s.
		X → M → Y	0.047	0.023	2.07	0.038
Metacognition and Study	Concentration	X → Y	−0.056	0.039	−1.45	n.s.
		X → M → Y	−0.008	0.011	−0.73	n.s.
	Selection pf principal aspects	X → Y	−0.001	0.060	−0.11	n.s.
		X → M → Y	0.050	0.023	2.17	0.030
	Self–assessment	X → Y	0.086	0.037	2.34	0.021
		X → M → Y	0.008	0.013	0.60	n.s.
	Strategies of preparation for a test	X → Y	0.023	0.021	1.08	n.s.
		X → M → Y	0.025	0.011	2.27	0.023
	Metacognitive sensitivity	X → Y	−0.043	0.044	−0.98	n.s.
		X → M → Y	0.028	0.017	1.81	n.s.

*X → Y, direct effect after entering the indirect effect; X → M → Y, indirect effect. No significant result (either indirect or direct effect) were pointed out in mediational models about “Motivation to study” “Organization of personal work,” “Use of supports,” “Concentration,” and “Metacognitive sensitivity,” and for this reason their results were removed from the table*.

No significant result (either indirect or direct effect) were pointed out in mediational models about “Motivation to study” “Organization of personal work,” “Use of supports,” “Concentration,” and “Metacognitive sensitivity,” and for this reason their results were removed from Tables 3–5.

These results suggest the possible effects of a conception of learning “Internal attribution for success and failure” as a mediator of the relationship between scales of QMS with all the academic outcomes.

An overall summarizing of the indirect effects is reported in the diagram below (Graph 1). The sign “+” inserted above the arrows identifies significant relationships between the specific metacognitive strategies reported in the box to the left (“Active elaboration of scholastic material,” “Flexibility to study,” “Participation in classroom,” “Selection of principal aspects,” “Strategies of preparation to a test”), the conception of learning named “Internal attribution of own success and failure” and, finally, all academic outcomes. So, the presence of the two signs “+” together identifies a significant mediational linkage between that specific metacognitive strategies and the academic outcomes.

## Discussion

In this study on middle-school students, metacognitive skills/strategies, conceptions of learning, and academic outcomes were assessed. With the first aim, the association between academic outcomes both with metacognitive skills/strategies and conceptions of learning was tested. Regarding metacognitive skills/strategies, the results of the study show insignificant associations with students' academic outcomes, except for self-assessment which was associated with academic outcomes in language and literature. Studies in literature showed the controversial nature of the relationship between metacognitive skills/strategies and academic outcomes. The results of the study are in line with previous studies suggesting inconsistent relationships between metacognitive skills/strategies and academic outcomes. For example, similarly to Sperling et al. (2004) and Kitsantas et al. (2008), Leong and Bettens (2002), Fonteyne et al. (2017) showed that the variable of metacognition failed to contribute to the prediction of academic outcomes of university students.

The unique association detected was between academic outcomes and self-assessment, meant as the evaluations learners make about their current knowledge levels in a particular domain of knowledge. In the literature the role of self-assessment with respect to academic outcomes is debated. The results of this study are in contrast with the recent trend of moving away to consider self-assessment as an indicator of learning outcomes (see, i.e., Sitzmann et al., 2010). In fact, they lead us to think that in the age range considered, learners become more accurate in the evaluation of their level of knowledge and more accurate insights of their being learners, resulting positively associated with academic outcomes.

Regarding conceptions of learning, as expected, the results of this study show a differential impact of the pattern of conceptions of learning on academic outcomes.

As predicted, conceptions of learning characterized by a social-constructive view of learning and personal growth are positively associated with some academic outcomes. In line with the literature (see, Pinto et al., 2018), a conception such as “getting involved” in learning through discussion and comparison with peers, teachers, and culture in general as well as a conception characterized by personal significance and personal growth show a direct positive effect on some academic outcomes.

Unexpectedly, a conception of learning such as “internal attribution of own success and failure” was the only one to be associated with all the academic outcomes considered (language and literature, foreign language, and math). A conception of learning characterized by internal attribution can be considered as an indicator of personal involvement in learning (Vezzani et al., 2017). Previous predictive models of conceptions of learning and academic outcomes in middle school students did not show the predictive role of the conception characterized by internal attribution (see, i.e., Pinto et al., 2018). However, the link between internal attribution and academic outcomes has already been suggested by previous research. For example, internal attribution in learning is linked with a sense of control in learning (Elliott and Dweck, 1988) and mastery-oriented studying (Daniels et al., 2008) that in turn is implicated in learning outcomes (see, i.e., Houston, 2016).

Furthermore, despite our expectation, conceptions of learning characterized by an incremental and quantitative view of learning were not negatively associated with academic outcomes.

Considering the lacking association between metacognitive skills/strategies and academic outcomes, we proceeded with the second aim of this study. In line with this, a predictive model of metacognitive skills/strategies and academic outcomes was tested with conception of learning “internal attribution for own success and failure” as mediating variables. The results of the mediation analyses showed that the relationship between metacognitive skills and strategies and academic outcomes was fully explained by the concurrent conception of learning as “internal attribution for own success and failure.” Specifically, high levels of metacognitive skills and strategies, such as “active elaboration of scholastic material,” “flexibility to study,” “participation in the classroom,” “selection of principal aspects,” “selection of preparation for a test” were associated with an increase in conception of learning characterized by internal attribution, which in turn predicted high academic outcomes.

These results indicated that the ways in which middle-school students approach the learning process could pass through the way they conceptualize learning and their perceptions of themselves as learners. Internal attribution pertains to personal representations of themselves as learners owned by students that could represent a part of the more general learner self-concept.

A study by Burnett et al. (2003) refuted the view that conceptions of learning are the direct antecedents of approaches to learning in introducing the mediating role of learner self-concept. The results of this study suggest intending the relationship between metacognitive skills and strategies and conceptions of learning, meant as part of the self-concept, in a reciprocal way rather than assuming a simplistic unidirectional direction.

In following this reasoning, the results of this study strengthen the thinking of Muis (2007) to intend the relationship between metacognition and conceptions of learning in a recursive way.

In summary, this study integrates the state-of-knowledge about the relationships between metacognitive skills/strategies and academic outcomes. Assuming a still yet ascertained influence of conceptions of learning on academic outcomes through metacognitive skills/strategies (Vermunt, 2005), these findings add a further piece of knowledge that is the further influence of metacognitive skills/strategies on academic outcomes through a particular conception of learning characterized by internal attribution. Although a direct effect of metacognitive skills and strategies on academic outcomes did not emerge in this sample of middle-school students with the exception of self-awareness, an indirect effect of metacognitive skills and strategies on academic outcomes through the mediation of a conception as internal attribution for success and failure emerged.

The identification of the mediating effect of this conception of learning leads to consider several implications for teaching and learning.

In considering desirable academic outcomes, the reciprocal influence of metacognitive skills/strategies and conceptions of learning should be considered. In the scholastic context, both variables need to be recognized in their value and both supported. The results of this study indicate that to reach successful academic outcomes, it may not be sufficient to be equipped with a metacognitive apparatus of study skills and strategies, but it is also necessary to develop an adequate representation of oneself as learner characterized by internal attribution of one's own success and failure, which means mastery of the process of learning with personal involvement.

Overall, the results of the present study constitute the first step from which to depart and construct further investigations including the possibility of replicating this study in other scholastic grades and educational contexts. Future research is necessary to disentangle relationships between conceptions of learning, metacognition, and academic outcomes.

Concerning the limitations of this study, as suggested by Foerst et al. (2017), the correlation between students' self-assessment of their strategies and application of their statements in learning situations is not always assured. In order to overcome this risk, future studies should consider the integration of data retrieved by different methods, for example reports and observations. In addition, in reviewing literature, differences regarding boy and girls' metacognitive skills emerged in several studies (see, i.e., Zimmerman and Martinez-Pons, 1990; Liliana and Lavinia, 2011), even if with incongruences (see, i.e., Jenkins, 2018). These discrepancies in results, deserve a deeper investigation to which further researches need to address. Finally, future studies should also investigate the reciprocal relationship between students' conceptions of learning and theory of mind. Past studies emphasized how task-related variables influence students' mentalization processes (see, Pinto et al., 2016) which, in turn, may influence the creation of conceptions of learning. Finally, future studies should also investigate whether students' conceptions of learning can be improved through modeling activities. Past studies have repeatedly demonstrated that specific prompts (e.g., individual writing, Bigozzi et al., 2011) or learning environments (peer-assisted learning, Tarchi and Pinto, 2015) can influence students' conceptualization, and this effect might transfer to conceptions of learning.

## Author contributions

All authors listed have made a substantial, direct and intellectual contribution to the work, and approved it for publication.

### Conflict of interest statement

The authors declare that the research was conducted in the absence of any commercial or financial relationships that could be construed as a potential conflict of interest.
